# Research on multi-channel access strategy based on congestion control with burst traffic in CRNs

**DOI:** 10.1371/journal.pone.0337319

**Published:** 2026-01-28

**Authors:** Qianyu Xu, Qi Zhang, Yang Bi, Jaafar Gaber

**Affiliations:** 1 Xi’an Aeronautical University, Xi’an, China; 2 Université de Technologie Belfort-Montbeliard, Belfort, France; Beijing Institute of Technology, CHINA

## Abstract

This paper investigates a multi-channel access strategy for cognitive radio networks (CRNs) under bursty traffic conditions, with a focus on congestion control. The proposed approach integrates cross-layer factors including channel fading, user activity, and finite cache capacity and models heterogeneous burst service arrivals using a two-state Markov-modulated Bernoulli process (MMBP-2). A dual-threshold mechanism is implemented in the node buffer to effectively manage congestion. System states are mapped onto a two-dimensional discrete Markov chain, where state transitions are characterized by a high-dimensional transition matrix. Through steady-state analysis, key performance metrics such as average queue length, throughput, delay, and packet loss rate are derived. Simulation results confirm that the model achieves stable operational performance. Building upon this framework, this paper proposes a multi-channel access strategy that maximizes average throughput while minimizing packet loss rate by employing a genetic algorithm. The results show that, in comparison with traditional strategies, the burst flow control model developed in this study effectively meets data access requirements in highly bursty environments. Furthermore, simulation experiments explore how system performance varies with changes in the number of channels and cognitive users, and the key operational threshold is determined. These findings offer valuable guidance for channel access design and capacity planning in burst communication scenarios.

## 1. Introduction

Smart city initiatives have increased demand for wireless communication services [1]. Conventional spectrum allocation system struggles to satisfy user demands, resulting in the growing scarcity of spectrum resources [2]. As a pivotal technology for enhancing the accessibility and reliability of 5G communication services [3–5], cognitive radio (CR) significantly improves spectrum utilization by enabling opportunistic spectrum access while ensuring no interference with primary users (PUs) [6]. As a transformative solution for wireless communications, CR technology has been extended to B5G/6G networks and Reconfigurable Intelligence Surface (RIS)-based communications. Key innovations, including channel hopping [7,8], user localization, and spectrum access, play a critical role in driving the development of Cognitive Radio Networks (CRNs), with channel access control garnering significant research interest.

However, the spectrum access strategy, which relies solely on spectrum sensing, has certain limitations. Providing a stable access environment for CRs represents a highly challenging task [9]. Literature [10] emphasized that the sensing results of CRs are influenced by factors such as path loss and channel fading in wireless links, while system configurations like link mechanisms and cache capabilities significantly impact the quality of service (QoS) for CRs [11]. In other words, the optimal access strategy can be obtained by evaluating the impact of environmental conditions and system configurations on spectrum resources, thereby facilitating resource integration.

Cross-layer resource integration enables flexible configuration of cognitive radio networks (CRNs), which is essential for optimizing the QoS in CRNs [12]. To achieve this, Literature [13] proposed a multi-channel access strategy that integrates factors such as path loss, channel fading, Automatic Repeat Request (ARQ), and finite buffer. Inspired by [13], Literature [14] further developed a QoS evaluation model by considering the access mode of multi-type burst services and multiple factors such as channel fading, user activities and buffer capacity.

Quantitative assessment of user service quality constitutes an indispensable approach for evaluating and ensuring network service performance [15]. However, user-centric wireless communication services, such as B5G/6G, typically encompass a variety of data services. Service data packets are transmitted in the form of service flows, which inherently exhibit burst characteristics. These bursts often lack distinct patterns and are challenging to capture accurately, while limited buffering capacity can cause network congestion or deadlock. Therefore, realistic traffic models should account for both burstiness and potential congestion issues.

Traditional channel access modeling frequently employs the Poisson distribution to characterize the arrival process of aggregated flows in an attempt to capture their batch arrival features [16]. However, the smooth Poisson process fails to adequately capture burstiness and correlation, resulting in substantial prediction inaccuracies. Meanwhile, traffic simulation using the Poisson model tends to result in overly idealized models, thereby placing the network in an excessively conservative state. Traffic modeling and analysis using queuing theory demand deeper investigation into service arrival distributions. Literature [17] suggested that establishing correlations among the burst sources can effectively simulate the arrival process of burst traffic flows, thereby advancing research on burst traffic modeling. Literature [18] proposed using MMBP to construct a martingale model for characterizing traffic arrival behavior, thus demonstrating the effectiveness of MMBP in addressing the challenge of heterogeneous service deployment.

The bursty nature of aggregated traffic frequently leads to network congestion issues. The implementation of threshold control in the buffer not only mitigates congestion but also implicitly reflects the arrival behavior of traffic, thereby contributing to predictive control. Literature [19] developed a discrete time Markov chain (DTMC) model to simulate the arrival behavior using a two-state MMBP. By setting threshold within the buffer and integrating closed-loop feedback control, the study demonstrated the effectiveness of threshold control in managing system performance. Literature [20] employed the MMBP model to analyze the burstiness and correlation in aggregated traffic, highlighting the interaction between congestion and bursty characteristics of traffic. Literature [21] demonstrates that, compared with traditional single-threshold setting, applying dual thresholds in the buffer can predict network congestion in advance and implement differentiated control based on service levels, thereby significantly enhancing the flexibility and stability of the system.

The presence of burst traffic undoubtedly brings unpredictability to the network. Although it has been demonstrated that addressing the channel access problem using the Markov model in an unknown environment is effective [22], as the intensity of data traffic bursts increases, the overall performance of the secondary network may exhibit a downward trend. Therefore, addressing the access issue of secondary users without ensuring system stability is considered impractical. This study addresses the dual challenges of burst traffic simulation and network congestion control, and by integrating multiple cross-layer parameters, ensures that the constructed model is more consistent with actual network characteristics. Furthermore, on the basis of evaluating the stability of the system, the optimal multi-channel access strategy is proposed. The main contributions are outlined as follows:

- *Integrated Multi-Layer Modeling for Burst Traffic Access and Congestion Control.* We investigate the access behavior of CRs under bursty traffic conditions, explicitly addressing network congestion through the integration of key cross-layer factors—channel fading, user activity, and finite buffer capacity. The proposed model is aligned with contemporary communication paradigms and provides a realistic characterization of CRNs operations in burst environments.
- *Mathematical Reformulation and Performance Analysis via Markov Modeling.* The bursty traffic control problem in CRNs is mathematically reformulated using a two-state Markov- Modulated Bernoulli Process (MMBP-2) to model the arrival process of heterogeneous burst services. System states are mapped onto a two-dimensional discrete Markov chain, and under dual-threshold congestion control, state transitions are represented through high-dimensional matrices. This analytical framework enables the derivation of key steady-state performance metrics for secondary systems such as average queue length, throughput, delay, and packet loss rate, thereby providing a practical foundation for designing multi-channel access strategies tailored to burst environments.
- *Genetic Algorithm-Optimized Multi-Channel Access Strategy.* We propose a novel multi- channel access strategy based on the analyzed access process. Simulations confirm stable performance of the proposed model, which incorporates key influencing factors. Leveraging this stability, we formulate a multi-objective optimization problem aimed at maximizing throughput and minimizing packet loss, and use a genetic algorithm (GA) to find optimal access solutions across diverse burst environments. This approach outperforms conventional strategies, especially under high burst intensity, with flexible objective function tuning to meet practical communication requirements.

## 2. System model

**Fig 1:**
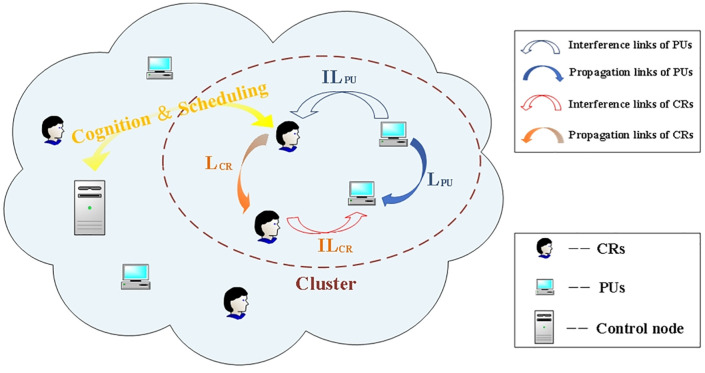
System model and propagation links.

In Fig 1, CRNs comprise M primary users (PUs) and N cognitive users (CRs), forming a single-cluster propagation link with time slots. Each cluster includes a control node for centralized data scheduling. PUs can only communicate via their designated licensed channel, assuming all channels have equal bandwidth. CRs periodically perform spectrum sensing within a time slot and can establish a connection only when the PU is not detected as using the channel. The control node can systematically manage multiple types of CRs traffic, assuming that CRs exhibit heterogeneity and are independently and identically distributed (i.i.d).

In time-slotted CRNs, each time slot (TS) is divided into two phases: the perception phase and the transmission phase. At the beginning of each TS, spectrum sensing is performed periodically, and the resulting perceptual information is broadcast to all cluster members via a dedicated channel. On this basis, the control node evaluates the probability of accessing each available channel and determines whether to utilize it for communication. Subsequently, data packets are transmitted during the remaining portion of the time slot. As depicted in Fig 1, the set of inter-cluster propagation links includes both propagation links and interference links, $\text{L}=\left\{{\text{L}}_{\text{PU}},{\text{L}}_{\text{CR}},\text{I}{\text{L}}_{\text{PU}},\text{I}{\text{L}}_{\text{CR}}\right\}$. Specifically, ${\text{L}}_{\text{PU}}$ and $\text{I}{\text{L}}_{\text{PU}}$ represent propagation links and interference links of PUs, while ${\text{L}}_{\text{CR}}$ and ${\text{IL}}_{\text{CR}}$ denote propagation links and interference links of CRs. This paper focuses exclusively on the uplink scenario, with analogous considerations applicable to the downlink.

## 3. Access process with burst traffic control

This section aims to develop a mathematical model for the access behavior of burst traffic in the secondary system. During this process, we will perform a thorough analysis of the characteristics of burst traffic and its implications for system performance, while also taking into account potential network congestion problems that may arise. To effectively tackle congestion issues, we will incorporate congestion control mechanisms into the node buffer, thereby ensuring the stability and efficient operation of the system.

Firstly, compared with regular stable traffic, burst traffic exhibits higher levels of unpredictability and volatility, thereby imposing a more intense demand on network resources. Secondly, burst traffic may cause network congestion, thereby significantly degrading system performance. Therefore, these potential risks must be taken into consideration when designing the access model. This section introduces a congestion control mechanism into the node buffer during the construction of the access model. Accordingly, the cognitive radio access process based on burst traffic consists of two components: access process of CRs and burst traffic control process.

### 3.1. Access process of CRs

**Fig 2:**
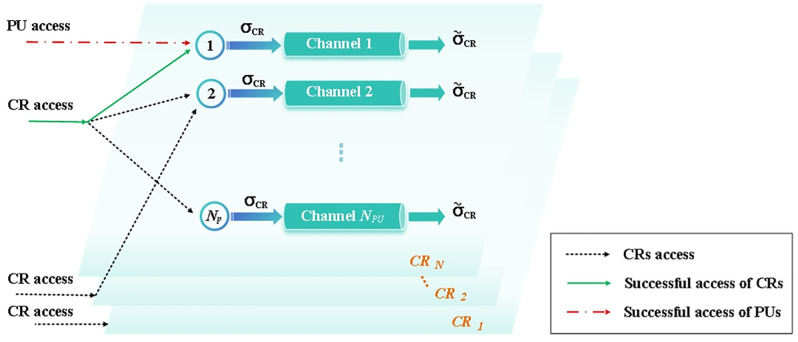
Access process of CRs.

Fig 2 illustrates the access process of CRs. During this process, CRs are not required to determine which PU owns the channel. In certain situations, CRs may opportunistically access the channel concurrently with the PU due to detection failure (for example, PUs and CRs on channel 1). If successful, they can exclusively utilize the channel for data transmission (such as CRs accessing channel 2). According to the sensing results obtained by CRs, we define the detection probability, ${\text{P}}_{\text{d}}$, is that CRs correctly identify the occupancy behavior of PUs. The false alarm probability, ${\text{P}}_{\text{f}}$, refers to the likelihood that CRs erroneously perceive a channel as occupied when, in fact, the PUs are not utilizing it. It is assumed that even in the event of a detection failure, CRs will be capable of maintaining communication without causing interference with PUs’ transmission. Based on this, two scenarios are considered:

- Case 1: Successful detection of CRs.

In this case, PUs do not transmit on the channel. Assuming that the radio link between any pair of nodes is considered to be affected by an independent random Nakagami flat fading channel, with the received channel envelope denoted as $r_{L_{CR}}$. Based on the relationship between the Nakagami-m distribution and the Gamma distribution [14], the successful acceptance probability, $P_{\left\{CRsucc|CR\;\right\}}$, can be determined by the SNR threshold in this cases where only CRs are present. It can be obtained that:


$$P_{\left\{CRsucc|CR\right\}}=\text{Pr}\;\left\{{\left|r_{L_{CR}}(t)\right|}^{\text{2}}>\frac{C_{CR}R_{L_{CR}}}{P_{CR}}\right\}=\frac{\Gamma (m_{L_{CR}},\;\frac{m_{L_{CR}}C_{CR}R_{L_{CR}}}{P_{CR}})}{\Gamma (m_{L_{CR}})}$$ (1)

Where ${\text{R}}_{{\text{L}}_{\text{CR}}}$ is the path loss of link ${\text{L}}_{\text{CR}}$, ${\text{m}}_{{\text{L}}_{\text{CR}}}$ is the Nakagami fading parameter of link ${\text{L}}_{\text{CR}}$, ${\text{C}}_{\text{CR}}$ and ${\text{P}}_{\text{CR}}$ are the receiving SNR and transmission power of CRs respectively. The calculation of the gamma function can be obtained by the $\Gamma (\text{n})=(\text{n}-\text{1})!,\;\Gamma (\text{n},\text{x})=(\text{n}-\text{1})!{\text{e}}^{-\text{x}}\sum_{\text{j}=\text{0}}^{\text{n}-\text{1}}\frac{{\text{x}}^{\text{j}}}{\text{j}!}$.

However, in the case of successful detection, denoted as $\sigma_{CR}$ for pre-channel acceptance, the probability depends not only on link propagation characteristics but also on the dynamic activities of PUs. The analysis of CRs assumes that all virtual packets are generated from the PUs and that PUs possess their own physical queue in each channel, denoted as${\;\text{K}}_{\text{PU}}$. Consequently, $\sigma_{CR}$ can be determined by both the queue length distribution of PUs and successful acceptance probability of CRs.


$$\sigma_{CR}=\text{Pr}\;\left\{{\text{K}}_{\text{PU}}\right\}\cdot P_{\left\{CRsucc|CR\right\}}\cdot (\text{1}-P_{f})$$ (2)

Where Pr$\left\{{\text{K}}_{\text{PU}}\right\}$ is the queue length distribution of PUs. According to the spectrum resource sharing mode, the physical queue length of the $\text{PU}\;({\text{K}}_{\text{PU}})$ is zero when there only CRs exist. Therefore, it can be concluded that,


$$\text{Pr}\left\{{\text{K}}_{\text{PU}}\right\}=\text{Pr}\;\left\{{\text{K}}_{\text{PU}}=\text{0}\right\}=\text{1}$$ (3)

Formula (3) represents that when only CRs exist in the channel, the probability of $\left\{{\text{K}}_{\text{PU}}=\text{0}\right\}$ is 1. In this case, the pre-channel acceptance probability of CRs before it enters the channel is,


$$\sigma_{CR}=P_{\left\{CRsucc|CR\right\}}\cdot (\text{1}-P_{f})$$ (4)

- Case 2: Failed detection of CRs

When the detection of CRs is failed, PUs and CRs will coexist in the channel. Let $P_{\left\{CRsucc|PU,\;CR\right\}}$ denote the successful acceptance probability, the probabilities can be formed as Pr{X>A+BY}, and the following results can be obtained [13].


$$P_{\left\{CRsucc|PU,\;CR\right\}}=\text{Pr}\;\left\{{\left|r_{L_{CR}}(t)\right|}^{\text{2}}>\frac{C_{CR}R_{L_{CR}}}{P_{CR}}+\frac{C_{CR}{P_{PU}R}_{L_{CR}}}{P_{CR}R_{IL_{PU}}}\cdot {\left|r_{IL_{PU}}(t)\right|}^{\text{2}}\right\}=\Phi (\frac{C_{CR}R_{L_{CR}}}{P_{CR}},\;\frac{C_{CR}{P_{PU}R}_{L_{CR}}}{P_{CR}R_{IL_{PU}}})$$ (5)

Let $\text{A}=\frac{C_{CR}R_{L_{CR}}}{P_{CR}},\;B=\frac{C_{CR}{P_{PU}R}_{L_{CR}}}{P_{CR}R_{IL_{PU}}}$, the following relationship can be derived from the gamma function.


$$\Phi (\text{A},\;\text{B})={\left(\frac{m_{Y}}{m_{X}B+m_{Y}}\right)}^{m_{Y}}e^{-m_{X}A}\sum_{n=\text{0}}^{m_{X}-\text{1}}\sum_{k=\text{0}}^{n}\frac{m_{X}^{n}B^{k}A^{n-k}\Gamma (m_{Y}+k)}{k!(n-k)!{\left(m_{X}B+m_{Y}\right)}^{k}\Gamma (m_{Y})}$$ (6)

Where $m_{X}$ can be any positive integer, $m_{Y}$ can be any real number. $R_{IL_{PU}}$ is the path loss of link $\text{I}{\text{L}}_{\text{PU}}$, $P_{PU}$ is the transmission power of the PU.

In this case, the pre-channel acceptance probability of CRs can be determined as follows.


$$\sigma_{CR}=\text{Pr}\;\left\{{\text{K}}_{\text{PU}}\neq \text{0}\right\}\cdot P_{\left\{CRsucc|PU,\;CR\right\}}\cdot (\text{1}-P_{d})$$ (7)

The principle of random advantage [23] states that terminals lacking packets in the buffer are able to transmit hypothetical virtual packets. It is easy to know that the physical queue length of the PUs is non-zero, that is $\text{Pr}\left\{{\text{K}}_{\text{PU}}=\text{0}\right\}=\text{0}$. According to $\text{Pr}\left\{{\text{K}}_{\text{PU}}\neq \text{0}\right\}=\text{1}-\text{Pr}\left\{{\text{K}}_{\text{PU}}=\text{0}\right\}$, the pre-channel acceptance probability of CRs can be simplified as follows.


$$\sigma_{CR}=P_{\left\{CRsucc|PU,\;CR\right\}}\cdot (\text{1}-P_{d})$$ (8)

Due to the impact of burst traffic, CRs are queued in the corresponding buffer prior to channel transmission, as illustrated by nodes 1 and 2 in Fig 2. Assume that the packet arrivals follow a Bernoulli distribution, and the sensing results will not affect the arrival rate of CRs. The probability of CR accessing channel i (i=1, 2,..., M) is denoted as ${\text{e}}_{\text{CR}-{\text{N}}_{\text{i}}}$. Given its capability to access any allocated channel, we can deduce that $\sum_{\text{i}=\text{0}}^{\text{M}}{\text{e}}_{\text{CR}-{\text{N}}_{\text{i}}}=\text{1}$. In the node buffer, burst traffic congestion control model will be implemented using a dual-threshold mechanism.

### 3.2. Burst traffic control process

**Fig 3:**
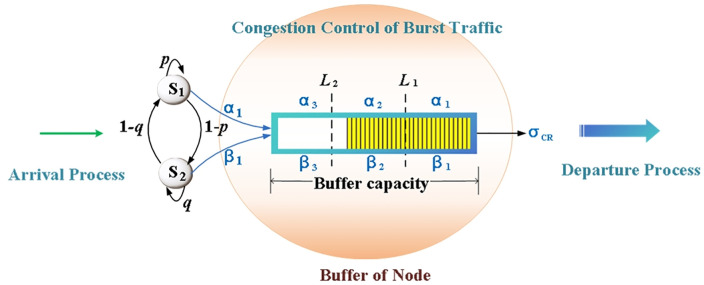
Burst traffic control process in node buffer.

Due to the diversity of signal sources, burst traffic entering the buffer exhibits varying arrival intensities. A two-state Markov-modulated Bernoulli process (MMBP-2) is employed to simulate the arrival process of two service types with distinct burst characteristics, including voice/video services and data/image services, and their correlations are established. The control process is shown in Fig 3. When data packets are queued in the buffer, congestion control is implemented using dual thresholds. Influenced by the access probability, the pre-channel acceptance probability of CRs $\left(\sigma_{CR}\right)$ differs from the departure rate after receiving the channel service (denoted as $\tilde{\sigma_{CR}}$). Therefore, the burst traffic control process in the buffer consists of two parts: arrival of burst traffic and congestion control.

#### 3.2.1. Arrival process of burst traffic.

Fig 3 simulates the arrival process of two types of service using MMBP-2, where state $S_{\text{1}}$ indicates data/image services, while state $S_{\text{2}}$ is voice/video services. The arrival rate is varies with the state transitions of a two-state Markov chain. In each state, packets arrive according to the Bernoulli distribution, where $\alpha_{\text{1}}$ and $\beta_{\text{1}}$ represent the arrival rates of states $S_{\text{1}}$ and $S_{\text{2}}$ respectively. There is a time-varying rate correlation between the two types of services. The transition probability p is utilized to establish this association when there is a burst in the data flow of a specific type of service.


$$\text{P}=[\begin{array}{cc} p & \text{1}-p\\ \text{1}-q & q \end{array}],\;\Lambda_{\text{1}}=[\begin{array}{cc} \alpha_{\text{1}} & \text{0}\\ \text{0} & \beta_{\text{1}} \end{array}]$$ (9)

By configuring the four statistical parameters $\left(\rho ,\;{\text{c}}^{\text{2}},\varphi ,\Phi (\text{x})\right)$ of MMBP-2, diverse sets of quad-tuple parameters $\left(\alpha_{\text{1}},\;\beta_{\text{1}},\;\text{p},\;\text{q}\right)$ can be determined to describe the correlation between two types of burst services with different arrival intensities. The statistical parameters of MMBP can be calculated by the following formulas [14].

- The average packet arrival rate of the two states ρ is:

$$\rho =\frac{\overset{―}{q}\alpha_{\text{1}}+\overset{―}{p}\beta_{\text{1}}}{\overset{―}{p}+\overset{―}{q}},\;\text{where}\;\overset{―}{p}=\text{1}-p,\;\overset{―}{q}=\text{1}-q$$ (10)

- The inter-arrival time’s squared coefficient of variation, denoted as ${\text{c}}^{\text{2}}$, characterizes the level of burstiness in the traffic generated by SUs.

$${\text{c}}^{\text{2}}=\frac{\text{2}\rho [{\left(\overset{―}{p}+\overset{―}{q}\right)}^{\text{2}}+(\overset{―}{p}\alpha_{\text{1}}+\overset{―}{q}\beta_{\text{1}})(\text{p}+\text{q}-\text{1})]}{\left(\overset{―}{p}+\overset{―}{q})[\overset{―}{q}\alpha_{\text{1}}+\overset{―}{p}\beta_{\text{1}}+\alpha_{\text{1}}\beta_{\text{1}}(p+q-\text{1})\right]}-\rho -\text{1}$$ (11)

- The first-order autocorrelation coefficient of the interarrival time, φ(1), while the x-th order auto-correlation coefficient of the number of SU’s arrivals based on MMBP-2, Φ(x). The two variables capture the correlation between SUs traffic arrivals.

$$\varphi (\text{1})=\;\frac{\alpha_{\text{1}}\beta_{\text{1}}{\left(\alpha_{\text{1}}-\beta_{\text{1}}\right)}^{\text{2}}\overset{―}{p}\overset{―}{q}{\left(p+q-\text{1}\right)}^{\text{2}}}{{\text{c}}^{\text{2}}{\left(\overset{―}{p}+\overset{―}{q}\right)}^{\text{2}}{\left[\overset{―}{q}\alpha_{\text{1}}+\overset{―}{p}\beta_{\text{1}}+\alpha_{\text{1}}\beta_{\text{1}}(p+q-\text{1})\right]}^{\text{2}}}$$ (12)

$$\Phi (\text{x})=\;\frac{{\left(\alpha_{\text{1}}-\beta_{\text{1}}\right)}^{\text{2}}\overset{―}{p}\overset{―}{q}{\left(p+q-\text{1}\right)}^{x}}{\left(\overset{―}{q}\alpha_{\text{1}}+\overset{―}{p}\beta_{\text{1}})[\overset{―}{q}({\text{1}-\alpha }_{\text{1}})+\overset{―}{p}(\text{1}-\beta_{\text{1}})\right]}$$ (13)

#### 3.2.2. Congestion control of burst traffic.

The congestion control of burst traffic is illustrated in Fig 3. In this approach, the minimum threshold ${\text{L}}_{\text{1}}$ and the maximum threshold ${\text{L}}_{\text{2}}$ are set in the buffer ($\text{0}<{\text{L}}_{\text{1}}<{\text{L}}_{\text{2}}<\text{K},$ while K represents the upper limit of the buffer capacity). Upon the arrival of a data packet,

- Case 1: If the current queue length is less than ${\text{L}}_{\text{1}}$, the data packet will be stored in the cache;
- Case 2: If the queue length is between ${\text{L}}_{\text{1}}$ and ${\text{L}}_{\text{2}}$, and the arrival process is in state ${\text{S}}_{\text{1}}$, the packet will be dropped with probability ${\text{1}-\alpha }_{\text{2}}$ to prevent possible congestion; if it is in state ${\text{S}}_{\text{2}}$, the packet will be dropped with probability $\text{1}-\beta_{\text{2}}$.
- Case 3: If the queue length exceeds ${\text{L}}_{\text{2}}$, and the arrival process is in state ${\text{S}}_{\text{1}}$, the packet will be dropped with probability ${\text{1}-\alpha }_{\text{3}}$; if it is in state ${\text{S}}_{\text{2}}$, the packet will be dropped with probability $\text{1}-\beta_{\text{3}}$.

In fact, the arrival rate of MMBP-2 can be effectively reduced through threshold control, which contributes to a greater mitigation of network congestion. In other words, the control model for burst traffic adjusts the arrival rate by employing dual thresholds. Let $\alpha_{\text{3}}<\alpha_{\text{1}},\;\beta_{\text{3}}<\beta_{\text{1}}$, then the packet sending rates $\alpha_{\text{m}},\;\beta_{\text{m}\;}(\text{m}=\text{1},\;\text{2},\;\text{3})$ of state ${\text{S}}_{\text{1}}$ and ${\text{S}}_{\text{2}}$ can be determined by the arrival rates in both states and the thresholds set in the buffer.


$$\alpha_{\text{m}}=\left\{ \begin{array}{c} \alpha_{\text{1}},\;\;\;\;\;\;\;\;\;\;\;\;\;\;\;\;\;\text{0}\leq \text{j}<{\text{L}}_{\text{1}}\\ \alpha_{\text{2}},\;\;\;\;\;\;\;\;\;\;\;\;\;\;\;\;{\text{L}}_{\text{1}}\leq \text{j}<{\text{L}}_{\text{2}}\\ \alpha_{\text{3}},\;\;\;\;\;\;\;\;\;\;\;\;\;\;\;\;{\text{L}}_{\text{2}}\leq \text{j}\leq \text{K} \end{array}\right.\;$$ (14)

$$\beta_{\text{m}}=\left\{ \begin{array}{c} \beta_{\text{1}},\;\;\;\;\;\;\;\;\;\;\;\;\;\;\;\;\;\text{0}\leq \text{j}<{\text{L}}_{\text{1}}\\ \beta_{\text{2}},\;\;\;\;\;\;\;\;\;\;\;\;\;\;\;\;{\;\text{L}}_{\text{1}}\leq \text{j}<{\text{L}}_{\text{2}}\\ \beta_{\text{3}},\;\;\;\;\;\;\;\;\;\;\;\;\;\;\;\;{\text{L}}_{\text{2}}\leq \text{j}\leq \text{K} \end{array}\right.\;$$ (15)

Where ${\alpha_{\text{2}}=\alpha }_{\text{1}}+\frac{\left(\alpha_{\text{3}}-\alpha_{\text{1}})(j-L_{\text{1}}+\text{1}\right)}{L_{\text{2}}-L_{\text{1}}+\text{1}}$, ${\beta_{\text{2}}=\beta }_{\text{1}}+\frac{\left(\beta_{\text{3}}-\beta_{\text{1}})(j-L_{\text{1}}+\text{1}\right)}{L_{\text{2}}-L_{\text{1}}+\text{1}}$.

### 3.3. Modeling and analysis of burst traffic control

According to queuing theory, the data packets stored in the buffer are regulated by the arrival process and the predefined threshold. Therefore, the data packet control process in the node buffer prior to channel access can be modeled as a two-dimensional discrete Markov chain, wherein the MMBP-2 serves as the traffic arrival model and the dual-threshold RED control as the traffic management mechanism. Suppose state $\left({\text{S}}_{\text{n}},\;\text{j}\right)$ represents the current state of the packet within the time slot; the system state can then be characterized using this Markov chain along with the buffer queue length as follows:


$$\left\{({\text{S}}_{\text{n}},\;\text{j}),\;\text{n}=\text{1},\;\text{2};\;\text{j}=\text{0},\;\text{1},\;\text{2},\;...,\;{\text{L}}_{\text{1}},\;...,\;{\text{L}}_{\text{2}},\;...,\text{K}\right\}$$ (16)

**Fig 4:**
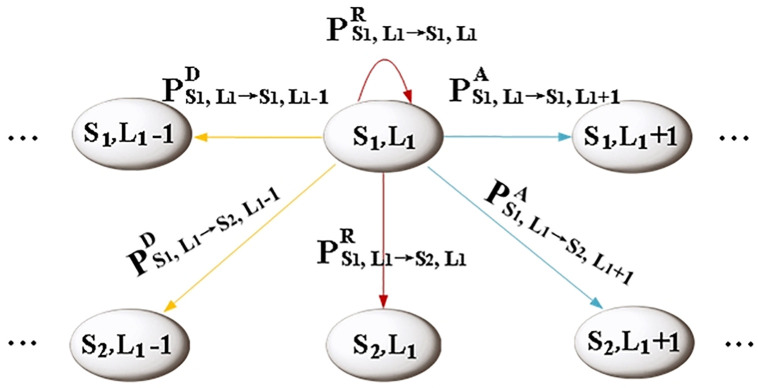
All possible transition scenarios in a given state.

As illustrated in Fig 4, we use state $\left({\text{S}}_{\text{1}},\;{\text{L}}_{\text{1}}\right)$ as an example to present all possible transition scenarios in a given state of the Markov chain, in order to analyze the transition probability. It is assumed that at most one data packet can arrive per time slot. From the perspective of buffer queue length, data packets may remain at the current level, ${\text{L}}_{\text{1}}$, in the next time slot if no new packets arrive. Alternatively, they may transition to the next queue length level, ${\text{L}}_{\text{1}}+\text{1}$, due to the arrival of new packets, or to the previous level, ${\text{L}}_{\text{1}}-\text{1}$, as packets are dequeued for channel service. Furthermore, as a binary relationship has been defined for identical queue length levels based on the burst traffic characteristics of MMBP-2, a one-step transition between ${\text{S}}_{\text{1}}$ and ${\text{S}}_{\text{2}}$ is evident.

Therefore, when the data packet is in state $\left({\text{S}}_{\text{1}},\;{\text{L}}_{\text{1}}\right)$, there are two scenarios for its one-step transition probability of the packet remaining in the current queue length state in the next time slot, ${\text{P}}_{\text{S}\;\to \text{L}}^{\text{R}}$. let ${\text{P}}_{{\text{S}}_{\text{1},\;}{\text{L}}_{\text{1}\;}\to {\text{S}}_{\text{1},\;}{\text{L}}_{\text{1}}}^{\text{R}}$ denote the probability of remaining in the state $\left({\text{S}}_{\text{1}},\;{\text{L}}_{\text{1}}\right)$, and Let ${\text{P}}_{{\text{S}}_{\text{1},\;}{\text{L}}_{\text{1}\;}\to {\text{S}}_{\text{2},\;}{\text{L}}_{\text{1}}}^{\text{R}}$ denote the probability that the packet transmission rate changes in the next time slot while the queue length remains constant. Correspondingly, we assume that the probabilities of the packet transitioning to ${\text{L}}_{\text{1}}+\text{1}$ and ${\text{L}}_{\text{1}}-\text{1}$ in the next time slot are ${\text{P}}_{\text{S}\;\to \text{L}}^{\text{A}}$ and ${\text{P}}_{\text{S}\;\to \text{L}}^{\text{D}}$, respectively. There are also four associated cases (in Fig 4): ${\text{P}}_{{\text{S}}_{\text{1},\;}{\text{L}}_{\text{1}\;}\to {\text{S}}_{\text{1},\;}{\text{L}}_{\text{1}\;}+\text{1}}^{\text{A}}$, ${\text{P}}_{{\text{S}}_{\text{1},\;}{\text{L}}_{\text{1}\;}\to {\text{S}}_{\text{2},\;}{\text{L}}_{\text{1}\;}+\text{1}}^{\text{A}}$, ${\text{P}}_{{\text{S}}_{\text{1},\;}{\text{L}}_{\text{1}\;}\to {\text{S}}_{\text{1},\;}{\text{L}}_{\text{1}\;}-\text{1}}^{\text{D}}$ and ${\text{P}}_{{\text{S}}_{\text{1},\;}{\text{L}}_{\text{1}\;}\to {\text{S}}_{\text{2},\;}{\text{L}}_{\text{1}\;}-\text{1}}^{\text{D}}$.

Based on the queue length state, combined with the arrival rate matrix $\Lambda_{m}=[\begin{array}{cc} \alpha_{m} & \text{0}\\ \text{0} & \beta_{m} \end{array}],\;m=(\text{1},\;\text{2},\;\text{3})$ and transition probability matrix $\text{P}=[\begin{array}{cc} p & \text{1}-p\\ \text{1}-q & q \end{array}]$, it can be deduced by transferring the situation that:


$$\left\{ \begin{array}{c} \begin{array}{c} {\text{P}}_{{\text{S}}_{\text{1},\;}{\text{L}}_{\text{1}\;}\to {\text{S}}_{\text{1},\;}{\text{L}}_{\text{1}\;}}^{\text{R}}=[\alpha_{\text{2}}\sigma_{CR}+({\text{1}-\alpha }_{\text{2}})(\text{1}-\sigma_{CR})]p\\ {\text{P}}_{{\text{S}}_{\text{1},\;}{\text{L}}_{\text{1}\;}\to {\text{S}}_{\text{2},\;}{\text{L}}_{\text{1}\;}}^{\text{R}}=[\alpha_{\text{2}}\sigma_{CR}+({\text{1}-\alpha }_{\text{2}})(\text{1}-\sigma_{CR})](\text{1}-p)\\ {\text{P}}_{{\text{S}}_{\text{1},\;}{\text{L}}_{\text{1}\;}\to {\text{S}}_{\text{1},\;}{\text{L}}_{\text{1}\;}+\text{1}}^{\text{A}}=\alpha_{\text{2}}p(\text{1}-\sigma_{CR}) \end{array}\\ \begin{array}{c} {\text{P}}_{{\text{S}}_{\text{1},\;}{\text{L}}_{\text{1}\;}\to {\text{S}}_{\text{2},\;}{\text{L}}_{\text{1}\;}+\text{1}}^{\text{A}}=\alpha_{\text{2}}(\text{1}-p)(\text{1}-\sigma_{CR})\\ {\text{P}}_{{\text{S}}_{\text{1},\;}{\text{L}}_{\text{1}\;}\to {\text{S}}_{\text{1},\;}{\text{L}}_{\text{1}\;}-\text{1}}^{\text{D}}=({\text{1}-\alpha }_{\text{2}})\sigma_{CR}p\\ {\text{P}}_{{\text{S}}_{\text{1},\;}{\text{L}}_{\text{1}\;}\to {\text{S}}_{\text{2},\;}{\text{L}}_{\text{1}\;}-\text{1}}^{\text{D}}=({\text{1}-\alpha }_{\text{2}})\sigma_{CR}(\text{1}-p) \end{array} \end{array}\right.\;$$

Thus, the transition matrix can be derived as follows:


$${\text{Q}}_{\text{2}(\text{K}+\text{1})\times \text{2}(\text{K}+\text{1})}=\begin{array}{cc} \left[\begin{array}{ccccccccccccc} E_{\text{1}}^{\prime } & F_{\text{1}}^{\prime } & & & & & & & & & & & \\ {\;D}_{\text{1}} & E_{\text{1}} & F_{\text{1}} & & & & & & & & & & \\ & \cdots & \cdots & \cdots & & & & & & & & & \\ & & {\;D}_{\text{1}} & E_{\text{1}} & F_{\text{1}} & & & & & & & & \\ & & & {\;D}_{\text{2}} & E_{\text{2}} & F_{\text{2}} & & & & & & & \\ & & & & {\;D}_{\text{2}} & E_{\text{2}} & F_{\text{2}} & & & & & & \\ & & & & & \cdots & \cdots & \cdots & & & & & \\ & & & & & & {\;D}_{\text{2}} & E_{\text{2}} & F_{\text{2}} & & & & \\ & & & & & & & {\;D}_{\text{3}} & E_{\text{3}} & {\;F}_{\text{3}} & & & \\ & & & & & & & & {\;D}_{\text{3}} & E_{\text{3}} & {\;F}_{\text{3}} & & \\ & & & & & & & & & \cdots & \cdots & \cdots & \\ & & & & & & & & & & {\;D}_{\text{3}} & E_{\text{3}} & {\;F}_{\text{3}}\\ & & & & & & & & & & & {\;D}_{\text{3}} & E_{\text{3}}^{\prime } \end{array}\right] & \begin{array}{c} \text{0}\\ \text{1}\\ \cdots \\ {\text{L}}_{\text{1}}-\text{1}\\ {\text{L}}_{\text{1}}\\ {\text{L}}_{\text{1}}+\text{1}\\ \cdots \\ {\text{L}}_{\text{2}}-\text{1}\\ {\text{L}}_{\text{2}}\\ {\text{L}}_{\text{2}}+\text{1}\\ \cdots \\ \text{K}-\text{1}\\ \text{K} \end{array} \end{array}$$ (17)

Where ${\text{F}}_{\text{1}}^{\prime }=\Lambda_{\text{1}}\text{P},\;{\text{E}}_{\text{1}}^{\prime }={(\text{I}-\Lambda }_{\text{1}}textrmP,\;{\;\text{D}}_{\text{m}}=\sigma_{\text{CR}}{(\text{I}-\Lambda }_{\text{m}})\text{P},\;{\text{E}}_{\text{m}}=\Lambda_{\text{m}}\sigma_{\text{CR}}\text{P}+(\text{I}-\Lambda_{\text{m}})(\text{1}-\sigma_{\text{CR}})\text{P},{\text{F}}_{\text{m}}=(\text{1}-\sigma_{\text{CR}})\Lambda_{\text{m}}\text{P},\;{\text{E}}_{\text{3}}^{\prime }=\Lambda_{\text{3}}\text{P}+({\text{I}-\Lambda }_{\text{3}})(\text{1}-\sigma_{\text{CR}})\text{P}$, while I is an identity matrix.

Due to the limited buffer capacity, Q is a layer-dependent finite-state quasi-birth-and- death matrix of size 2(K+1)×2(K+1), with each element being a square matrix of dimension 2×2. The transfer matrix Q indicates that each state can receive two types of burst traffic.

### 3.4. Steady state and performance metrics

In CRNs, the research on access strategy without considering system stability lacks practical significance. Consequently, this section analyzes the stability of the system model and evaluates the performance metrics of the secondary system under steady-state conditions, thereby providing a basis for subsequent research.

The steady-state of the secondary system at any queue length state j, is determined by two types of tuples with distinct burst levels.


$$\pi_{j}=\pi (\text{1},\;j)+\pi (\text{2},\;j)$$

Combining the steady-state equation, πQ=π, and the normalization condition, πe=1, the steady-state solution vector $\vec{\pi }=(\pi_{\text{0}},\;\pi_{\text{1}},\;\pi_{\text{2}},\;...,\;\pi_{\text{K}})$ can be obtained. Briefly speaking, since the state space of the Q-matrix is two-dimensional, computing the steady-state probability vector becomes a high-dimensional problem that lacks an explicit analytical solution. However, it can be observed that the level structure of the Q-matrix depends on the configured buffer capacity K. Consequently, by specifying the value of K, the exact form of Q can be determined, allowing the steady-state solution to be computed in Matlab using level reduction algorithms [14] based on numerical analysis. Therefore, based on the steady-state distribution, $\vec{\pi }$, the performance metrics for the secondary system can be obtained as follows.

- The average queue length of the secondary system, denoted by E(L), reflects the long-term average number of CRs in the system’s queuing state. This value is determined by the established model and typically fluctuates around the buffer capacity.

$$\text{E}(\text{L})=\sum_{\text{j}=\text{0}}^{\text{K}}\text{j}\pi_{j}=\sum_{\text{j}=\text{0}}^{\text{K}}\text{j}[\pi (\text{1},\;j)+\pi (\text{2},\;j)]$$ (18)

- The average throughput of the secondary system, denoted by E(S), represents the average number of data packets that are successfully delivery from the secondary system to the channel per unit time. This value is primarily determined by the specific scenario, system design, and resource flexibility. Due to potential congestion caused by burst traffic, the throughput is typically reduced to 30% ~ 70% of the theoretical bandwidth under the effects of packet loss and retransmission. In this study, the results are expressed as a percentage directly related to the pre-channel acceptance probability during calculation.

$$\text{E}(\text{S})=\sigma_{CR}(\text{1}-\pi_{\text{0}})=\sigma_{CR}[\text{1}-\pi (\text{1},\;\text{0})-\pi (\text{2},\;\text{0})]$$ (19)

- The average delay of the secondary system, E(W), refers to the average duration from the arrival of a CR at the buffer to its departure from it, which is primarily determined by the average queue length and the average throughput. In a bursty environment, the delay may abruptly increase to 10–100 times the steady-state value, and packets may even be discarded due to queue overflow. In an elastic system with dynamic resource allocation, the steady-state delay typically ranges from 10 to 100 ms, while the burst delay falls within 100–500 ms.

$$\text{E}(\text{W})=\frac{\text{E}(\text{L})}{\text{E}(\text{S})}=\frac{\sum_{\text{j}=\text{0}}^{\text{K}}\text{j}\pi_{j}}{\sigma_{CR}(\text{1}-\pi_{\text{0}})}$$ (20)

- The packet loss rate of the secondary system, ${\text{D}}_{\text{pl}}$, denotes the probability that an arriving packet is rejected or discarded under steady-state conditions, which is determined by the proportion of packets that are not successfully served. Since the RED algorithm relies on the dynamic adjustment of the threshold, under normal scenarios, there exists ${\text{0}\leq \text{D}}_{\text{pl}}\leq {\text{D}}_{\text{pl}}^{\text{max}}$ (here it is usually assumed that ${\text{D}}_{\text{pl}}^{\text{max}}=\text{30}\%$). As network congestion approaches, this value may rapidly increase to ${\text{D}}_{\text{pl}}^{\text{max}}$, and in extreme scenarios, it can reach 100%.

$$D_{pl}=\pi_{K}(\text{1}-\sigma_{CR})=[\pi (\text{1},\;K)+\pi (\text{2},\;K)]\cdot (\text{1}-\sigma_{CR})$$ (21)

## 4. Multi-channel access strategy

In CRNs, it is impractical to study access scenarios in isolation from system stability. The performance of secondary system depends not only to the activities of PUs but also on the detection results of CRs. This section focuses on analyzing the impact of PU activities on the performance of CRNs when CRs succeed or fail in detection, and proposes a multi-channel access strategy on the basis of verifying system stability. The general parameters involved in this study are set as follows in Table 1 to obtain more reasonable simulation results.

**Table 1:** The general parameter setting.

Parameter	Value	Parameter	Value
$m_{L_{CR}}$	1	$P_{f}$	0.05
$C_{CR}$	6	$P_{d}$	0.8
$R_{L_{CR}}$	5	$P_{PU}$	24
$R_{{IL}_{PU}}$	18	$P_{CR}$	20
${\text{L}}_{\text{1}}$	4	${\text{L}}_{\text{2}}$	11
K	15	$N_{P}$	5

### 4.1. Impact of PUs’ activity on the performance of secondary system

The stability of the secondary system is validated in this section, establishing a foundational basis for access strategy research. The burst scenarios are simulated by adjusting the four statistical parameters of MMBP-2, $\left(\rho ,\;{\text{c}}^{\text{2}},\varphi ,\Phi (\text{x})\right)$, and their settings follow general rules (see literature [14]). Five traffic flows (Traffic 1 to Traffic 5) with different burst intensities are simulated by setting the statistical parameters. The arrival rates of the two states can be calculated according to Formulas (10)-(13), as shown in Table 2. It can be observed from the arrival rate that the burst intensity gradually decreases from Traffic 1 to Traffic 5.

**Table 2:** Five traffic flows with different burst intensities.

Traffic	ρ	$c^{2}$	φ	Φ	$\alpha_{1}$	$\beta_{1}$
Traffic 1	0.5	50	0.1	0.8	0.90671	0.001824
Traffic 2	0.5	100	0.2	0.7	0.852653	0.001665
Traffic 3	0.5	150	0.3	0.6	0.80139	0.00151
Traffic 4	0.5	200	0.4	0.5	0.750796	0.00134
Traffic 5	0.5	250	0.5	0.4	0.700459	0.001147

**Fig 5:**
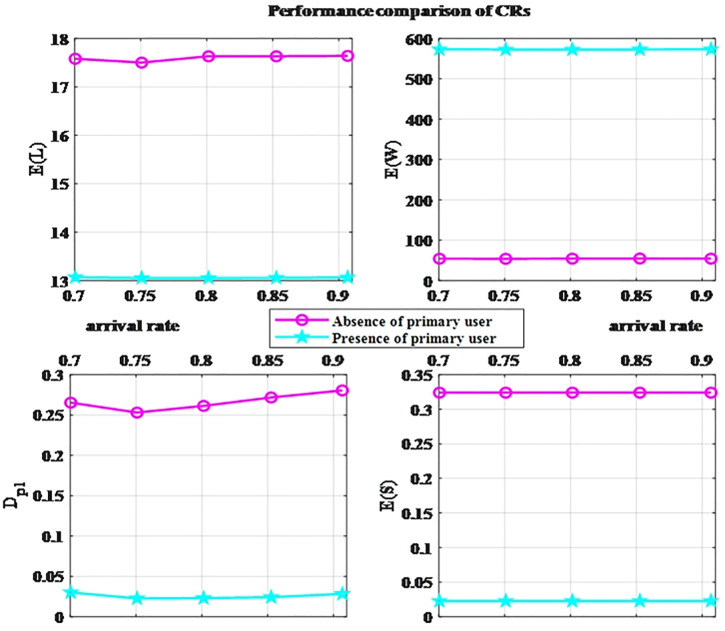
Performance comparison of CRs.

If CRs successfully detect the PU, there will be no PUs in the system; if the detection fails, they will coexist with the PUs on the same channel. As can be seen from the previous analysis, the pre-channel acceptance probability of CRs, $\sigma_{\text{CR}}$, in these two scenarios is different. In Fig 5, we present a comparison of the performance metrics of the CRs in the two scenarios. According to the comparison with the benchmark of performance indicators (in section 3.4), although there is a significant difference in performance results between the two scenarios, both remain within an acceptable error range. Moreover, under different arrival rates (i.e., different burst intensities), the curves are relatively stable, which proves that the research model established in this study can ensure the stable operation performance of the secondary network regardless of whether PUs are present.

### 4.2. Multi-channel access strategy based on burst traffic control

Under the premise of system stability, this section investigates the multi-channel access strategy of CRs based on genetic algorithm (GA). GA relies on a population-based search mechanism and can effectively escape local optima while proactively selecting conservative access strategies through mutation operations, thereby avoiding interference to PUs. Meanwhile, this algorithm inherently supports multi-objective optimization, enabling it to provide highly adaptive access strategies according to different scenarios and flexibly balance various conflicting requirements. Considering the throughput limitation caused by burst traffic and the packet loss problem induced by congestion control, this paper takes average throughput and packet loss rate as the main optimization objectives.

- Objective 1: Optimization of E(S), noted as ${\text{v}}_{\text{s}}(\text{e})$. When CRs access a specific channel, the average throughput based on the access probability can be determined using formula (19):

$${\text{v}}_{\text{s}}(\text{e})=\sigma_{CR}(\text{1}-\pi_{\text{0}})\cdot e_{CR-N_{i}}$$ (22)

- Objective 2: Optimization of ${\text{D}}_{\text{pl}}$, noted as ${\text{v}}_{\text{d}}(\text{e})$. After accessing to a specific channel, the packet loss rate based on the access probability is obtained using formula (21):

$${\text{v}}_{\text{d}}(\text{e})=\pi_{K}(\text{1}-\sigma_{CR})\cdot e_{CR-N_{i}}$$ (23)

The optimal channel access strategy should maximize E(S) while minimizing ${\text{D}}_{\text{pl}}$. The model is:


$$\text{P}-\text{1}:\;{\text{v}}_{\text{s}}^{*}(\text{e})=\text{arg}\;\text{max}\;{\text{v}}_{\text{s}}(\text{e});\;\text{s}.\text{t}.\;:\;\text{0}\leq e_{CR-N_{i}}\leq \text{1}\;\text{and}\;\sum_{\text{i}}e_{CR-N_{i}}=\text{1}$$ (24)

$$\text{P}-\text{2}:\;{\text{v}}_{\text{d}}^{*}(\text{e})=\text{arg}\;\text{min}\;{\text{v}}_{\text{d}}(\text{e});\;\text{s}.\text{t}.\;:\;\text{0}\leq e_{CR-N_{i}}\leq \text{1}\;\text{and}\;\sum_{\text{i}}e_{CR-N_{i}}=\text{1}$$ (25)

The procedural flow for implementing the multi-channel access strategy of CRs based on GA is as follows.

**Table:** 

Multi-channel Access Strategy of CRs Based on Genetic Algorithm		
***•***	***Given***	
√	*M = 10*	*% Population size*
√	*G = 12*	*% maximum generation*
√	*P*_*c*_ * = 0.9*	*% crossover probability*
√	*P*_*m*_ * = 0.09*	*% mutation probability*
√	*R=*[*0,1*]	*% variation range*
√	*A = *0.00000001	*% searching precision*
√	*V*_*s*_ *(e), V*_*d*_ *(e)*	*% fitness function*
***•***	***Algorithms:***	
1:	***Procedure()***	
2:	// Population Initialization	
3:	To ensure the non-negativity of the fitness function, the objective functions of P-1, P-2 in Equations (21), (22) is transformed as follows.	
	Vs(e)=Vs*(e)+Cmin	Vd(e)=-Vd*(e)+Cmax
	*Cmin* - denotes a predetermined smaller value.	
	*Cmax* - denotes a predetermined larger value.	
4:	Calculating the fitness function *Vs(e), Vd(e)*	
5:	Individuals are selected from population by proportional selection algorithm according to fitness value	
6:	if (random (0, 1) < *P*_*c*_)	
	do crossover operation according to *P*_*c*_	
	if (random (0, 1) < *P*_*m*_)	
	do mutation operation according to *P*_*m*_	
7:	Get a new population	
8:	Recording the best chromosomes	
9:	Until: Fitness value of any chromosome ≥ fitness function	
10:	or *G > 12*	
11:	End ***Procedure()***	

Eight traffic flows with different burst intensities are simulated, and their binary arrival rates are determined by the four statistical parameters of MMBP-2, $\left(\rho ,\;{\text{c}}^{\text{2}},\varphi ,\Phi (\text{x})\right)$, as shown in Table 3.

**Table 3:** Arrival rates of eight traffic flows with different burst intensities.

Traffic	${(\alpha }_{1},\beta_{1})$	Traffic	${(\alpha }_{1},\beta_{1})$
Traffic 1	(0.9067097119, 0.0018239742)	Traffic 5	(0.7004590849, 0.0011466865)
Traffic 2	(0.8526533347, 0.0016654833)	Traffic 6	(0.6502478001, 0.0009256213)
Traffic 3	(0.8013900590, 0.0015098426)	Traffic 7	(0.6001108086, 0.0006682983)
Traffic 4	(0.7507958849, 0.0013392691)	Traffic 8	(0.5500289350, 0.0003644730)

**Fig 6:**
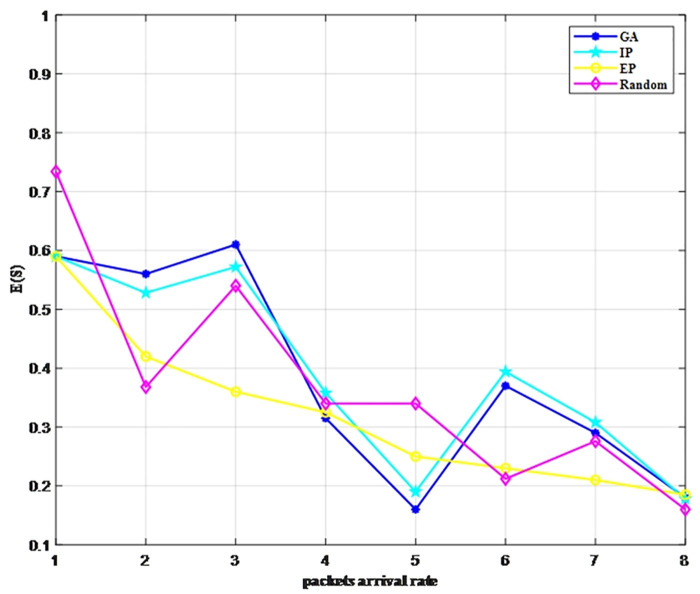
Comparison of E(S) under different strategies.

**Fig 7:**
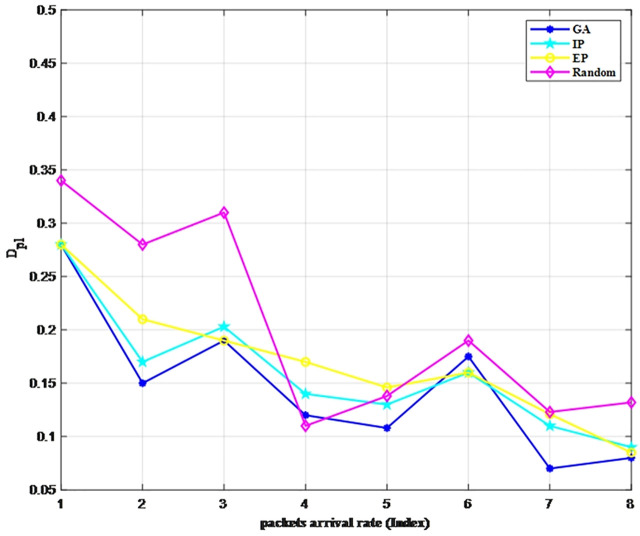
Comparison of D_pl_ under different strategies.

We use two metrics, E(S) and $D_{pl}$, which are set as the objective functions, to verify the access strategies under different burst intensities (Traffic 1 to Traffic 8). Figs 6 and 7 illustrate the advantages of our GA-optimized multi-channel access strategy over three conventional approaches: the equal probability (EP) method, inverse proportion (IP) strategy, and random selection (Random) strategy. The EP strategy means that the probability of accessing each channel is equal, denoted as $\frac{\text{1}}{N_{p}}$. The IP strategy sets the access probability of each channel as $\frac{\alpha_{\text{1}}}{\sum_{N_{p}}\alpha_{\text{1}}}$.

In general, as the burst intensity decreases, regardless of the access strategy adopted, both metrics decrease with the arrival rate. We can see that the GA strategy performs significantly better than the other three strategies in terms of average data transmission. Notably, its packet loss rate and throughput exhibit superior performance under conditions of high burst intensity. Moreover, under different burst intensities, the performance metrics of the IP strategy are very close to those of the GA strategy. This is because the time complexity of the GA strategy is higher than that of the IP strategy, resulting in more accurate outcomes. Consequently, the IP strategy can be considered an approximate solution for the online scheduling using the GA strategy, whereas for offline scenarios such as that addressed in this paper, the GA strategy can be appropriately applied to improve accuracy.

**Fig 8:**
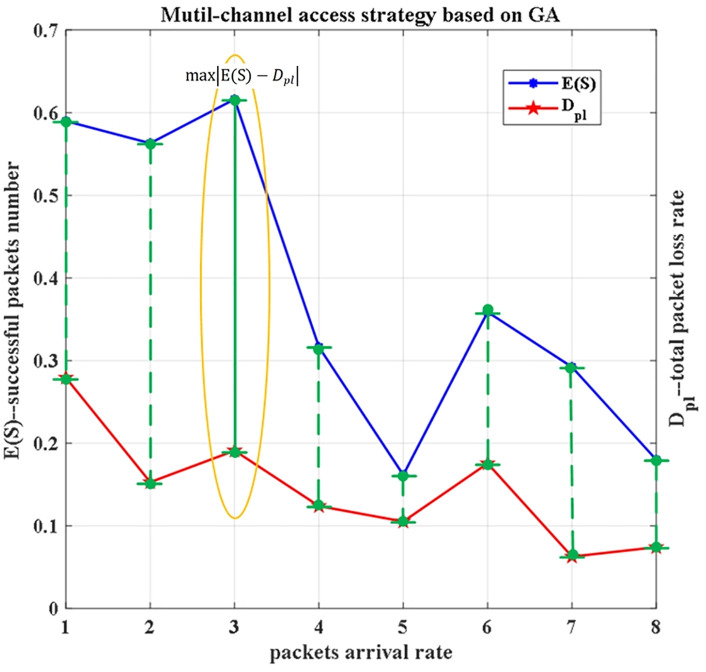
Multi-channel access strategy based on GA.

Next, we will analyze the optimal strategy of the GA under multiple iterations. According to the objective function we set, the multi-channel access strategy can achieve optimal system performance when E(S) is maximized and $D_{pl}$ is minimized. As shown in Fig 8, this occurs when burstiness is Traffic 3. At this time, there exists $\text{max}\left|\text{E}(\text{S})-D_{pl}\right|$, and the burstiness statistical parameters are ρ=0.5, ${\text{c}}^{\text{2}}=\text{150}$, φ(1)=0.3 and Φ(x)=0.6, which enables an optimal solution for system performance under this strategy.

The aforementioned research thoroughly investigates the access mechanism in scenarios involving burst traffic management, innovatively proposes a multi-channel access strategy, and derives the optimal solution for heterogeneous burst environments using genetic algorithms. This study provides significant theoretical basis for developing multi-channel access strategies within the proposed access framework. In practical applications, the objective function can be flexibly configured according to specific communication requirements, highlighting the adaptability of the established burst traffic control model.

### 4.3. System performance analysis

In this section, we analyze the variation of system performance under changes in the number of channels (or the number of PUs) and the number of users (or the number of CRs).

**Fig 9:**
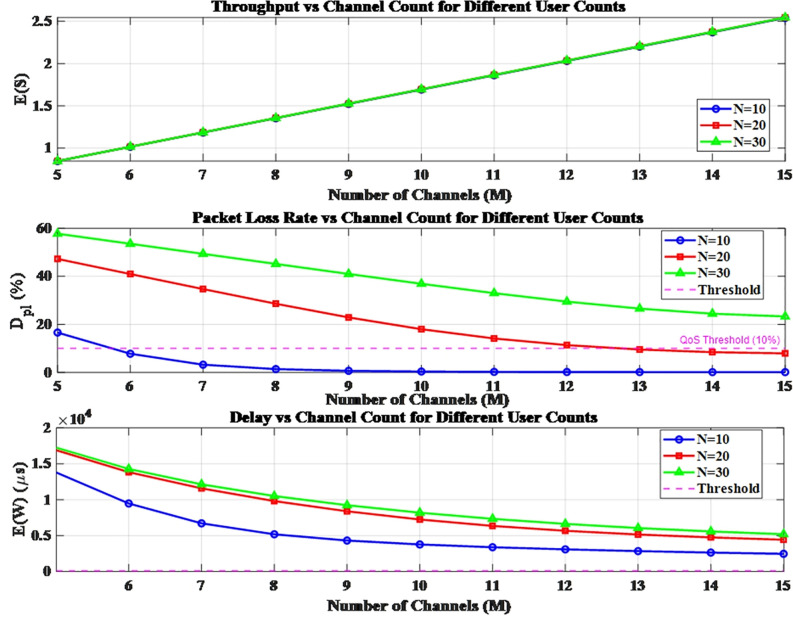
The variations under different user counts.

First, we conduct a parameter sensitivity analysis. In the burst scenario of Traffic 1, the number of channels (M) ranges from 5 to 15 in steps of 1, and the number of users (N) ranges from 10 to 30 in steps of 2. We illustrate the variations of the three metrics (E(S), E(W) and $D_{pl}$) under different user counts for varying channel counts (Fig 9). It can be observed from the figure that all three metrics fall within their respective benchmark ranges. In burst scenarios, the throughput (referring to the number of packets served per unit time) increases with more of the number of channels; in contrast, both the packet loss rate and delay decrease as the number of channels increases. Moreover, system performance improves when there are more channels and fewer users.

**Fig 10:**
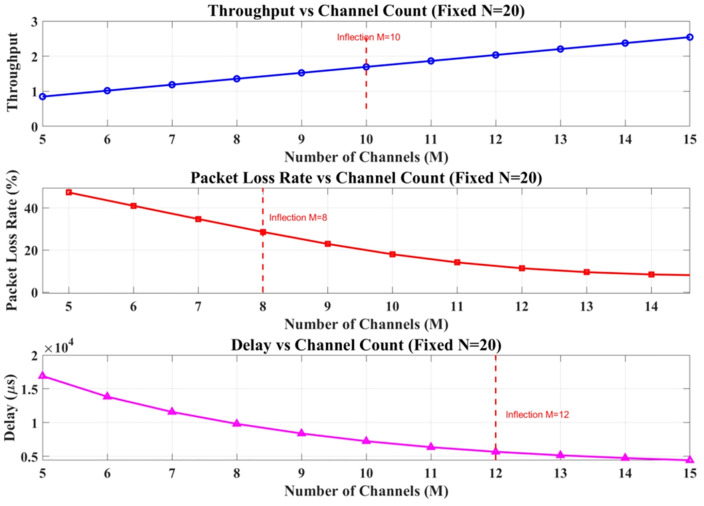
The variations when fixed N = 20.

Next, we analyze the network bottleneck under system scale expansion. As shown in Fig 10, when the number of channels is fixed at 10, variations in the number of users have a nonlinear impact on system performance. N = 20is identified as the critical load threshold: before this point, throughput increases linearly; beyond it, performance deteriorates sharply. Specifically, when N exceeds 20, the packet loss rate surges above 20% and continues to rise, while delay increasingly violates QoS requirements. QoS analysis reveals that the system enters an overload state when the number of users exceeds 22, suggesting that the optimal user capacity should be maintained within the range of 18–22.

**Fig 11:**
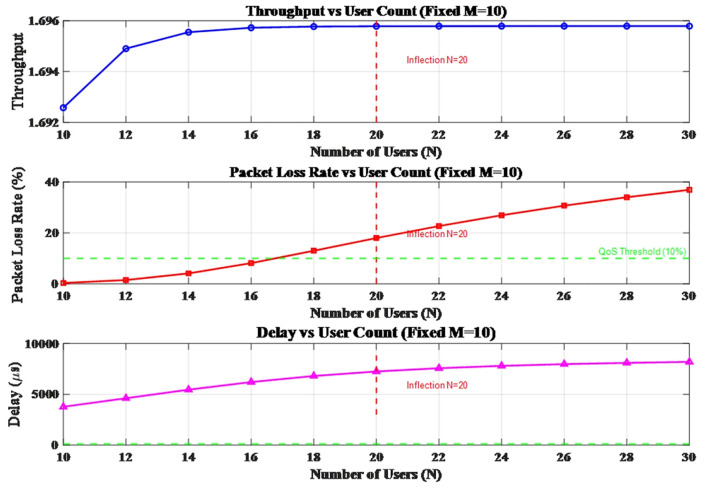
The variations when fixed M = 10.

As shown in Fig 11, when the number of users is fixed at 20, increasing the number of channels significantly enhances system performance. Throughput increases monotonically with the number of channels, showing a clear inflection point at M = 10: before this point, each additional channel contributes approximately a 15% increase in throughput, whereas the improvement rate declines to 5% afterward, indicating that system capacity is nearing saturation. The packet loss rate reaches as high as 32% when M = 5 but decreases rapidly with more channels, exhibiting another inflection point at M = 8, where the rate of decline is halved. At M = 10, the packet loss rate drops to 9.8%, satisfying the QoS requirement of 10%. The delay characteristic follows an exponential decay pattern, with the rate of reduction slowing notably after M = 12.

Furthermore, the experiment finds that the packet loss rate grows quadratically with the number of users. A comprehensive analysis demonstrates that for N = 20 users, M = 10 represents the optimal channel configuration, as it simultaneously meets the QoS thresholds for both packet loss rate and delay. The above research findings provide valuable guidance for capacity planning in bursty environments.

## 5. Conclusions

This paper focuses on a series of modeling studies regarding cognitive radio access issues in emergency environments, integrated with network congestion control. We established a burst flow control access model by incorporating multiple cross-layer factors and adopted the MMBP-2 as the input. Based on the two-dimensional discrete Markov process, the state transition matrix of the secondary system was constructed to derive the corresponding performance metrics. The calculation methods and statistical significance of these performance metrics are presented, and the stability of the proposed system is verified accordingly. On this premise, we discuss a multi-objective channel access strategy based on genetic algorithms with respect to throughput and packet loss rate, and elaborate on its implementation procedures. By comparing this algorithm with three other basic algorithms, its superiority is validated, and the optimal channel access scheme is proposed. In addition, we designed parameter sensitivity analysis experiments to investigate variations in system performance under different numbers of channels and users and determined the key operation thresholds. Simulation results demonstrate that the burst flow control model proposed in this paper enables adaptive multi-channel access strategies in bursty environments. Furthermore, our findings indicate that when the number of users is N = 20, setting the number of channels to M = 10 achieves optimal performance, as it satisfies the QoS requirements for both packet loss rate and delay. These research outcomes offer meaningful insights for capacity planning in dynamic and high-variability communication scenarios.

## References

[1] Sethi SK, Mahapatro A. Interference aware intelligent routing in cognitive radio based Vehicular Adhoc networks for smart city applications. Int J Inf Tecnol. 2023. doi: 10.1007/s41870-023-01434-0

[2] Prasad RK, Jaya T. Intelligent Spectrum Sharing and Sensing in Cognitive Radio Network by Using AROA (Adaptive Rider Optimization Algorithm). Int J Comp Intel Appl. 2023;22(01). doi: 10.1142/s1469026823410079

[3] Shruti KR. Channel allocation and ultra-reliable communication in CRNs with heterogeneous traffic and retrials: A dependability theory-based analysis. Computer Communications. 2020;158:51–63. doi: 10.1016/j.comcom.2020.04.055

[4] Dey S, Misra IS. Quality ensured non-orthogonal multiple access based downlink scalable video coding multicasting over cognitive radio network. Trans Emerging Tel Tech. 2021;33(1). doi: 10.1002/ett.4382

[5] Khan AU, Abbas G, Abbas ZH, Khan WU, Waqas M. Spectrum utilization efficiency in CRNs with hybrid spectrum access and channel reservation: A comprehensive analysis under prioritized traffic. Future Generation Computer Systems. 2021;125:726–42. doi: 10.1016/j.future.2021.07.024

[6] Yuan S, Zhang Y, Ma T, Cheng Z, Da Guo. Graph convolutional reinforcement learning for resource allocation in hybrid overlay–underlay cognitive radio network with network slicing. IET Communications. 2022;17(2):215–27. doi: 10.1049/cmu2.12527

[7] Sa S, Mahapatro A. Role-Based Channel Hopping Algorithm for a Cognitive Radio Network in Asynchronous Environment. Wireless Pers Commun. 2021;127(3):2083–102. doi: 10.1007/s11277-021-08771-y

[8] Sa S, Mahapatro A. A composite channel hopping algorithm for blind rendezvous in heterogeneous cognitive radio networks. Telecommun Syst. 2024;86(3):417–32. doi: 10.1007/s11235-024-01123-8

[9] Min S, Chunsheng X, Yanxiao Z, Xiuzhen C. Dynamic spectrum access: from cognitive radio to network radio. IEEE Wireless Commun. 2012;19(1):23–9. doi: 10.1109/mwc.2012.6155873

[10] Subrat KS, Arunanshu M, Nabanita M. Optimal resource allocation to improve energy efficiency of cognitive radio-based vehicular Ad Hoc network under imperfect sensing. Advances in Energy Technology. 2020:221–34.

[11] Jung E, Liu X. Opportunistic Spectrum Access in Multiple-Primary-User Environments Under the Packet Collision Constraint. IEEE/ACM Trans Networking. 2012;20(2):501–14. doi: 10.1109/tnet.2011.2164933

[12] Stamou A, Kakkavas G, Tsitseklis K, Karyotis V, Papavassiliou S. Autonomic Network Management and Cross-Layer Optimization in Software Defined Radio Environments. Future Internet. 2019;11(2):37. doi: 10.3390/fi11020037

[13] Zhang L, Song T, Hu J, Bao X. Analysis of Spectrum Access Strategy with Multiple Cross-Layer Considerations in Cognitive Radio Networks. Wireless Pers Commun. 2015;87(4):1383–400. doi: 10.1007/s11277-015-3067-x

[14] Xu Q, Li S, Do TV, Jia K, Yang N. Performance Analysis of Cognitive Radio Networks With Burst Dynamics. IEEE Access. 2021;9:110627–38. doi: 10.1109/access.2021.3103321

[15] Thien HT, Vu V-H, Koo I. A Transfer Games Actor–Critic Learning Framework for Anti-Jamming in Multi-Channel Cognitive Radio Networks. IEEE Access. 2021;9:47887–900. doi: 10.1109/access.2021.3068129

[16] Chydzinski A, Samociuk D, Adamczyk B. Burst ratio in the finite-buffer queue with batch Poisson arrivals. Applied Mathematics and Computation. 2018;330:225–38. doi: 10.1016/j.amc.2018.02.021

[17] Guan L, Awan IU, Woodward ME. Stochastic modelling of random early detection based congestion control mechanism for bursty and correlated traffic. IEE Proc, Softw. 2004;151(5):240. doi: 10.1049/ip-sen:20041089

[18] Chi XF, Sun YX, Yu BZ. SFC bandwidth-by-hop allocation and deployment based on Martingale theory for statistical delay QoS guarantee. Chinese Patent; 2022.

[19] Lim LB, Guan L, Grigg A, Phillips IW, Wang XG, Awan IU. Controlling mean queuing delay under multi-class bursty and correlated traffic. Journal of Computer and System Sciences. 2011;77(5):898–916. doi: 10.1016/j.jcss.2010.08.007

[20] Alsaaidah A, Zalisham M, Fadzli M, Abdel-Jaber H. Markov-Modulated Bernoulli-Based Performance Analysis for Gentle BLUE and BLUE Algorithms under Bursty and Correlated Traffic. Journal of Computer Science. 2016;12(6):289–99. doi: 10.3844/jcssp.2016.289.299

[21] Li S, Xu Q, Gaber J, Dou Z, Chen J. Congestion Control Mechanism Based on Dual Threshold DI-RED for WSNs. Wireless Pers Commun. 2020;115(3):2171–95. doi: 10.1007/s11277-020-07676-6

[22] Xu Z, Zhang Z, Wang S, Jolfaei A, Bashir AK, Yan Y, et al. Decentralized Opportunistic Channel Access in CRNs Using Big-Data Driven Learning Algorithm. IEEE Trans Emerg Top Comput Intell. 2021;5(1):57–69. doi: 10.1109/tetci.2020.3018779

[23] Rao RR, Ephremides A. On the stability of interacting queues in a multiple-access system. IEEE Trans Inform Theory. 1988;34(5):918–30. doi: 10.1109/18.21216

